# Case report: Variant-specific pre-exposure prophylaxis of SARS-CoV-2 infection in multiple sclerosis patients lacking vaccination responses

**DOI:** 10.3389/fimmu.2022.897748

**Published:** 2022-07-25

**Authors:** Christina Woopen, Urszula Konofalska, Katja Akgün, Tjalf Ziemssen

**Affiliations:** Center of Clinical Neuroscience, Department of Neurology, University Hospital Carl Gustav Carus Dresden, Technical University of Dresden, Dresden, Germany

**Keywords:** multiple sclerosis, sphingosine-1-receptor modulators (S1PR), neutralizing antibody, prophylaxis, severe acute respiratory syndrome coronavirus 2 (SARS-CoV-2), coronavirus disease (COVID-19), vaccination, case report

## Abstract

Sphingosine-1-phosphate receptor modulators and anti-CD20 treatment are widely used disease-modifying treatments for multiple sclerosis. Unfortunately, they may impair the patient’s ability to mount sufficient humoral and T-cellular responses to vaccination, which is of special relevance in the context of the SARS-CoV-2 pandemic. We present here a case series of six multiple sclerosis patients on treatment with sphingosine-1-phosphate receptor modulators who failed to develop SARS-CoV-2-specific antibodies and T-cells after three doses of vaccination. Due to their ongoing immunotherapy, lacking vaccination response, and additional risk factors, we offered them pre-exposure prophylactic treatment with monoclonal SARS-CoV-2-neutralizing antibodies. Initially, treatment was conducted with the antibody cocktail casirivimab/imdevimab. When the SARS-CoV-2 Omicron variant became predominant, we switched treatment to monoclonal antibody sotrovimab due to its sustained neutralizing ability also against the Omicron strain. Since sotrovimab was approved only for the treatment of COVID-19 infection and not for pre-exposure prophylaxis, we switched treatment to tixagevimab/cilgavimab as soon as it was granted marketing authorization in the European Union. This antibody cocktail has retained, albeit reduced, neutralizing activity against the Omicron variant and is approved for pre-exposure prophylaxis. No severe adverse events were recorded for our patients. One patient had a positive RT-PCR for SARS-CoV-2 under treatment with sotrovimab, but was asymptomatic. The other five patients did not develop symptoms of an upper respiratory tract infection or evidence of a SARS-CoV-2 infection during the time of treatment up until the finalization of this report. SARS-CoV-2-neutralizing antibody treatment should be considered individually for multiple sclerosis patients lacking adequate vaccination responses on account of their immunomodulatory treatment, especially in times of high incidences of SARS-CoV-2 infection.

## Introduction

Many disease-modifying drugs are available for the treatment of multiple sclerosis (MS). Selected immunomodulatory agents like sphingosine-1-phosphate receptor (S1PR) modulators and anti-CD20 treatment limit the patients’ ability to mount sufficient immune responses to vaccination (1). This is of special relevance in the context of the ongoing severe acute respiratory syndrome coronavirus 2 (SARS-CoV-2) pandemic.

MS patients at the MS Center Dresden, Germany, are screened for SARS-CoV-2-specific antibody and T-cellular responses after vaccination in order to detect those lacking an adequate immune response (2). Unfortunately, we found several patients under certain disease-modifying therapies who developed neither humoral nor T-cellular responses to SARS-CoV-2 vaccination, even after the application of three mRNA and/or vector vaccine doses. The lacking immune response to vaccination puts these patients at risk for contracting SARS-CoV-2 infection and for suffering a severe course of coronavirus disease (COVID-19). Additional factors like age > 50 years, cardiovascular disease, diabetes mellitus, obesity, chronic lung, kidney, or liver disease, can further increase the risk for severe COVID-19.

Fortunately, treatment options for patients who are not able to mount sufficient responses to active immunization are available. According to the recommendations of several German Medical Societies, passive immunization with neutralizing anti-SARS-CoV-2-antibodies as pre-exposure prophylactic treatment should be offered to those patients who have an increased risk for a severe course of COVID-19 due to immunosuppression (e.g. caused by hematooncological disease, immunotherapy, or hereditary immune defects) and who did not respond sufficiently to active immunization (3).

Here, we present a case series of six MS patients under immunomodulatory treatment who lacked antibody and T-cell responses to three SARS-CoV-2 vaccinations and who were thus prophylactically treated with SARS-CoV-2-neutralizing antibodies in our MS Center. In accordance with the prevalent SARS-CoV-2 variants at the time, treatment was started with casirivimab/imdevimab, subsequently switched to sotrovimab, and finally to tixagevimab/cilgavimab. To our knowledge, no cases of pre-exposure prophylactic SARS-CoV-2-neutralizing antibody treatment in MS patients have been reported yet.

### Casirivimab/imdevimab

Casirivimab/imdevimab (Ronapreve/REGEN-COV; Roche Registration GmbH) is a monoclonal human IgG1 antibody cocktail with neutralizing activity against SARS-CoV-2. Each antibody binds to a distinct epitope on the receptor binding domain of the viral spike protein. The two antibodies are used in combination in order to enhance efficacy in the face of emerging virus variants and to decrease the risk of selection for viral escape mutations.

The neutralizing antibody cocktail first received emergency use authorization in the USA in November 2020. In November 2021, the European Commission granted marketing authorization for casirivimab/imdevimab for the treatment of selected patients with COVID-19 disease and for the prophylaxis of SARS-CoV-2 infection in adults and adolescents aged 12 years and older weighing at least 40 kg (4). In the case of pre-exposure prophylaxis, the antibodies casirivimab and imdevimab are initially administered at doses of 600 mg each as a single intravenous infusion or *via* subcutaneous injection. As long as prophylaxis is needed, treatment is repeated every four weeks at a dose of 300 mg each (4).

First results from trials evaluating the clinical efficacy of casirivimab/imdevimab in the treatment of COVID-19 and in the prophylaxis of SARS-CoV-2 infection have been reported. Published data from an ongoing clinical trial demonstrated that treatment with the antibody cocktail reduced hospitalization and death rates of non-hospitalized COVID-19 patients compared to placebo (5). Furthermore, the treatment led to a faster resolution of symptoms and to an accelerated decrease in viral load (5). In another analysis of the same clinical trial, the reduction in SARS-CoV-2 viral load mediated by casirivimab/imdevimab was more pronounced in previously seronegative patients (6). The rate of adverse events was similar between casirivimab/imdevimab and placebo (5, 6).

Concerning prophylaxis of COVID-19, the risk of asymptomatic SARS-CoV-2-infected patients to develop symptomatic disease was decreased in the group receiving casirivimab/imdevimab compared to the group receiving placebo (7). Moreover, the neutralizing antibody cocktail was able to prevent asymptomatic and symptomatic SARS-CoV-2 infection in individuals living in a household with infected persons (8). In the study participants who did contract SARS-CoV-2 in this setting, casirivimab/imdevimab treatment abbreviated the time of symptomatic disease and of high viral load (8). Preliminary data from a phase 1, double-blind, placebo-controlled study evaluating the repeated application of subcutaneous casirivimab/imdevimab every four weeks showed a significant risk reduction for the development of COVID-19 compared to participants receiving placebo treatment while there was no difference in serious adverse events between the two groups (9).

Casirivimab/imdevimab demonstrated effective neutralization of the SARS-CoV-2 Delta variant *in vitro*. Problematically, it was shown to insufficiently neutralize the SARS-CoV-2 Omicron variant (10, 11).

### Sotrovimab

Sotrovimab (VIR-7831; GlaxoSmithKline/Vir Biotechnology) is an engineered human monoclonal IgG1 antibody produced in Chinese Hamster Ovary cells. It neutralizes SARS-CoV-2 by binding to a highly conserved epitope on the viral spike protein located outside of the receptor-binding motif.

Sotrovimab was granted marketing authorization by the European Commission in December 2021 for the treatment of adults and adolescents aged > 12 years and weighing more than 40 kg who suffer from COVID-19, do not need oxygen supplementation, and have an increased risk of developing a severe disease course (12). It is recommended to start the treatment within five days after symptom onset. The antibody is administered at a dose of 500 mg intravenously.

An ongoing double-blind phase 3 trial compared disease progression to hospitalization or death between outpatients receiving sotrovimab or placebo. The analyzed population comprised non-hospitalized adults with symptomatic COVID-19 and at least one risk factor for a severe disease course. Patients were eligible if symptoms had begun within the previous five days and if they had mild-to-moderate COVID-19. An interim analysis of this trial showed that treatment with sotrovimab led to a significant risk reduction for hospitalization and death in comparison to placebo (13). Adverse events were similar between groups receiving sotrovimab and placebo, severe adverse events were less common in sotrovimab-treated patients compared to the placebo group (13).

Results of the double-blind, randomized TICO (Therapeutics for Inpatients with COVID-19) trial showed that sotrovimab did not improve clinical outcomes in adults hospitalized due to COVID-19 (14).

Several *in vitro* studies were able to show that sotrovimab and its parent monoclonal antibody, S309, fully or largely retain their neutralizing capacity also against the SARS-CoV-2 Omicron variant (10, 15, 16).

### Tixagevimab/cilgavimab

Tixagevimab/cilgavimab (Evusheld/AZD7442; AstraZeneca AB) is a combination of two monoclonal antibodies with neutralizing activity against SARS-CoV-2 derived from B cells of SARS-CoV-2-infected persons. Modifications were added in order to prolong the antibodies’ half-life and to decrease binding of the Fc receptor and complement component C1q. The antibodies are produced in Chinese Hamster Ovary cells. They are directed against distinct, non-overlapping epitopes of the receptor binding domain of the SARS-CoV-2 spike protein (17).

Tixagevimab/cilgavimab received marketing authorization in the European Union in March 2022 for pre-exposure prophylaxis of COVID-19 in adults and adolescents aged 12 years and older weighing at least 40 kg (18). The antibodies are administered as two separate intramuscular injections at a dose of 150 mg each in a 1.5 mL solution. Median terminal elimination half-life was estimated to be 89 days for tixagevimab and 84 days for cilgavimab. Protection is expected to last at least six months after one dose of tixagevimab/cilgavimab based on data from the PROVENT (Phase 3 Study of Efficacy and Safety of AZD7442 for Pre-exposure Prophylaxis of COVID-19 in Adults) trial so that injections can be repeated every six months (18).

Data from the ongoing double-blind PROVENT study showed a significant risk reduction for symptomatic COVID-19 disease in participants treated with tixagevimab/cilgavimab as compared to placebo within a median follow-up period of 83 days (17). The study population comprised individuals with an increased risk for inadequate responses to active SARS-CoV-2 vaccination or with an increased risk of exposure. Incidences of severe adverse events were not different between treatment and placebo groups.

According to several *in vitro* studies, the combination of tixagevimab and cilgavimab retains neutralizing activity against SARS-CoV-2 Omicron variants, however at a reduced level as compared to previous SARS-CoV-2 strains (19–21). Two studies evaluated the neutralizing capacity of sera obtained from immunocompromised patients after treatment with the antibody cocktail. Bruel et al. found an efficient neutralization of the Delta variant for all patient sera, but a reduced neutralizing activity against Omicron (22). Benotmane et al. reported that less than 10% of the analyzed patient sera were able to neutralize the Omicron BA.1 variant. They suggested that the antibody dose is probably insufficient and may need to be adapted (23). Correspondingly, the duration of protection after one application of tixagevimab/cilgavimab is likely shorter for the Omicron than for the other SARS-CoV-2 variants (18).

## Case descriptions

The screening of patients at our MS Center in Dresden, Germany, for humoral and T-cellular responses to active SARS-CoV-2 immunization yielded several cases without detectable immune responses to three vaccine doses. For the respective patients, we evaluated the option of passive immunization with monoclonal SARS-CoV-2-neutralizing antibodies as pre-exposure prophylaxis.

Patient characteristics are summarized in **Table 1**. All reported patients received immunomodulatory treatment with S1PR modulators. Five of six patients had a diagnosis of relapsing-remitting MS (RRMS) and were treated with fingolimod, one received siponimod for therapy of secondary progressive MS. None of the patients had a history of suspected or confirmed status post SARS-CoV-2 infection before start of monoclonal antibody treatment.

**Table 1:** Patient characteristics.

Patient #	Age (y)	sex	MS type	DMT	EDSS	Comorbidities/other risk factors	1st/2nd vaccination date	Vaccine type	3rd vaccination date	Vaccine type	1st casirivimab/imdevimab	2nd casirivimab/imdevimab	1st sotrovimab	1st tixagevimab/cilgavimab
**1**	63	m	SPMS	SIP	6.0	normocytic anemia	22 Feb 2021	BNT162b2	30 Jul 2021	BNT162b2	16 Dec 2021	13 Jan 2022	10 Feb 2022	7 Apr 2022
							15 Mar 2021	BNT162b2						
**2**	54	m	RRMS	FTY	2.0	wife with breast cancer on chemo- and radiotherapy	5 Apr 2021	BNT162b2	21 Sep 2021	BNT162b2	17 Dec 2021	14 Jan 2022	11 Feb 2022	8 Apr 2022
							26 Apr 2021	BNT162b2						
**3**	50	m	RRMS	FTY	2.0	suspected arterial hypertension	15 May 2021	BNT162b2	4 Nov 2021	mRNA-1273	10 Jan 2022	none	7 Feb 2022	4 Apr 2022
							5 Jun 2021	BNT162b2						
**4**	65	f	RRMS	FTY	4.0	arterial hypertension; hypercholesterolemia; autoimmune thyroiditis; slight overweight	5 May 2021	BNT162b2	3 Dec 2021	mRNA-1273	10 Jan 2022	none	7 Feb 2022	4 Apr 2022
							9 Jun 2021	BNT162b2						
**5**	64	m	RRMS	FTY	2.0	basal cell carcinoma, excision Sep and Oct 2021; hypercholesterolemia	2 May 2021	AZD1222	23 Nov 2021	BNT162b2	12 Jan 2022	none	9 Feb 2022	6 Apr 2022
							29 Jun 2021	BNT162b2						
**6**	44	f	RRMS	FTY	1.5	3 school-age children	22 Mar 2021	AZD1222	23 Sep 2021	mRNA-1273	14 Jan 2022	none	11 Feb 2022	8 Apr 2022
							18 Jun 2021	BNT162b2						

y, years; m, male; f, female; MS, multiple sclerosis; SPMS, secondary progressive MS; RRMS, relapsing-remitting MS; DMT, disease-modifying treatment; SIP, siponimod; FTY, fingolimod; EDSS, Expanded Disability Status Scale.

**Figure 1:**
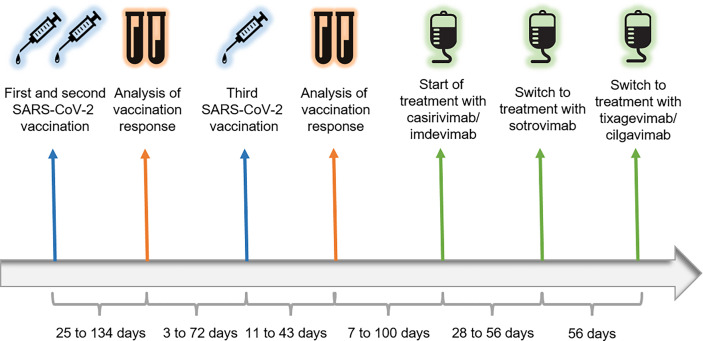
Timeline. Patients were vaccinated with two doses of SARS-CoV-2 mRNA and/or vector vaccine between February and June, 2021. Humoral and T-cellular responses to vaccination were analyzed 25 to 134 days after the second vaccine dose of each patient. All reported patients lacked SARS-CoV-2-specific antibodies and T-cells so that they received a third vaccination 3 to 72 days after the analysis of their immune response. After an interval of 11 to 43 days to the third vaccination, analysis of immune responses was repeated. Again, all patients did not have antibody and T-cellular responses to SARS-CoV-2. Neutralizing antibody treatment was discussed with the patients and initiated 7 to 100 days after the analysis. Initially, patients received infusions with casirivimab/imdevimab every four weeks. With rising incidences of infections with the SARS-CoV-2 Omicron variant, we switched treatment to sotrovimab 28 to 56 days after the first casirivimab/imdevimab infusion had taken place. As sotrovimab was formally approved only for the treatment of COVID-19 infection, we switched treatment to tixagevimab/cilgavimab as soon as it received marketing authorization in the European Union. This antibody cocktail is approved for pre-exposure prophylaxis of SARS-CoV-2 infection and has retained neutralizing capacity against the SARS-CoV-2 Omicron strain.

The temporal sequence of vaccinations, analyses of immune responses, and initiation of neutralizing antibody treatment is depicted in **Figure 1**. All patients received two doses of SARS-CoV-2 vaccine in the time between February and June, 2021. Four of them were vaccinated with two doses of BNT162b2 (BioNTech/Pfizer), two patients received a first dose of AZD1222 (Oxford-AstraZeneca) and a second dose of BNT162b2. Immune responses to vaccination were measured with an interval of at least 25 days after the second vaccine dose. Antibodies directed against the SARS-CoV-2 spike protein were initially measured *via* LIAISON^®^ SARS-CoV-2 S1/S2 IgG quantitative chemiluminescence immunoassay (DiaSorin). Values < 12.0 AU/mL were considered negative as indicated in the manufacturer’s instructions. From October 2021 onwards, antibodies were measured *via* LIAISON^®^ SARS-CoV-2 TrimericS IgG quantitative chemiluminescence immunoassay (DiaSorin). For this assay, values < 33.8 BAU/mL were classified as negative according to manufacturer’s information. T-cellular responses were measured *via* QuantiFERON^®^ SARS-CoV-2 assay (Qiagen). Here, interferon-gamma secretion of T-cells after 18 to 24 hours of stimulation with SARS-CoV-2 spike protein peptide pools 1 and 2 was measured *via* enzyme-linked immunosorbent assay. Values < 0.15 IU/mL were considered negative in line with manufacturer’s instructions. All reported patients showed negative SARS-CoV-2 spike-specific IgG antibodies and T-cellular responses after two doses of vaccination. Subsequently, all patients received a third SARS-CoV-2 vaccine dose. Three of them were vaccinated with BNT162b2, the other three with mRNA-1273 (Moderna). Control of vaccination responses took place 11 to 43 days after the third immunization and showed persistently negative antibody and T-cell responses to SARS-CoV-2 spike protein in all patients (**Table 2**).

**Table 2:** Antibody and T-cellular response to SARS-CoV-2 vaccination in patients 1 to 6.

Patient #	Analysis of antibody and T-cell response to SARS-CoV-2 after second vaccination	S protein- specific antibody response	T-cell response to antigen pools S1/S2 (IU/mL)	Analysis of antibody and T-cell response to SARS-CoV-2 after third vaccination	S protein- specific antibody response	T-cell response to antigen pools S1/S2 (IU/mL)
**1**	27 Jul 2021	<3.8 AU/mL	0	7 Sep 2021	4.31 AU/mL	0.0065
			0			0
**2**	9 Aug 2021	<3.8 AU/mL	0	19 Oct 2021	25.8 BAU/mL	0
			0			0.003
**3**	13 Sep 2021	<3.8 AU/mL	0	13 Dec 2021	7.65 BAU/mL	0
			0			0
**4**	5 Oct 2021	<4.81 BAU/mL	0	14 Dec 2021	<4.81 BAU/mL	0.0045
			0.0205			0.014
**5**	12 Oct 2021	<4.81 BAU/mL	0.0595	5 Jan 2022	7.43 BAU/mL	0
			0			0
**6**	13 Jul 2021	4.62 AU/mL	0	1 Nov 2021	12.5 BAU/mL	0
			0			0

AU/mL, Antibody Units per milliliter; BAU/mL, Binding Antibody Units per milliliter; IU/mL, International Units per milliliter. SARS-CoV-2-specific antibody and T-cell responses were measured after the second and again after the third vaccination of each patient. IgG antibodies against the spike protein of SARS-CoV-2 were measured via LIAISON^®^ SARS-CoV-2 S1/S2 IgG quantitative chemiluminescence immunoassay (DiaSorin; values < 12.0 AU/mL considered negative). From October 2021 onwards, antibodies were measured via LIAISON^®^ SARS-CoV-2 TrimericS IgG quantitative chemiluminescence immunoassay (DiaSorin; values < 33.8 BAU/mL considered negative). T-cellular interferon-gamma secretion to SARS-CoV-2 spike protein peptide pools 1 and 2 was measured via QuantiFERON^®^ SARS-CoV-2 assay (Qiagen; values < 0.15 IU/mL considered negative). All patients had negative antibody and T-cellular responses to SARS-CoV-2 spike protein after the second and third vaccination.

Patient 1 is a 63-year-old male receiving siponimod for the treatment of secondary progressive MS. His degree of neurological disability is relatively high with a score of 6.0 in the Expanded Disability Status Scale (EDSS). His laboratory examination shows normocytic anemia as comorbidity.

Patient 2 is male and 54 years old. He takes fingolimod for the treatment of RRMS and has a lower degree of disability with an EDSS value of 2.0. His wife has been diagnosed with breast cancer and is receiving chemo- and radiotherapy posing her at risk for a severe COVID-19 disease course.

Patient 3 is a 50-year-old male with an EDSS score of 2.0, receiving medication with fingolimod for therapy of RRMS. Arterial hypertension is suspected as a comorbidity, but has not yet been confirmed at the time of preparation of this manuscript.

Patient 4 is a female RRMS patient who is 65 years old and takes fingolimod. She has an EDSS score of 4.0 corresponding to a limitation of her walking range. She has several cardiovascular risk factors comprising arterial hypertension, hypercholesterolemia, and slight overweight. Furthermore, she suffers from autoimmune thyreoiditis.

Patient 5 is a 64-year-old male patient taking fingolimod for treatment of RRMS with an EDSS value of 2.0. He was operated on a facial basal cell carcinoma in September and October, 2021. His laboratory exam displays hypercholesterolemia as cardiovascular risk factor.

Patient 6 is a female RRMS patient with an age of 44 years taking fingolimod. She has a low value of 1.5 in the EDSS and no relevant comorbidities. She has three school-age children limiting her ability to reduce social contacts.

As casirivimab/imdevimab was granted marketing authorization for the prophylaxis of SARS-CoV-2 infection in adults by the European Commission in November 2021, we discussed this therapeutic option with eligible patients. The reported patients were in favor of a pre-exposure prophylactic treatment with the neutralizing antibodies and the first intravenous infusions with 600 mg each of casirivimab and imdevimab took place in our MS Center between 16^th^ of December, 2021, and 14^th^ of January, 2022. Patients 1 and 2 received their second casirivimab/imdevimab infusion at a dose of 300 mg each on January 13^th^ and 14^th^, 2021, respectively. No severe adverse events occurred in our patients. Patient 1 and 6 reported chills and fatigue after the first casirivimab/imdevimab infusion. The former further reported more frequent occurrence of dizziness and headache than usual during the weeks up until the second infusion. During the first casirivimab/imdevimab infusion, hypertensive blood pressure was measured in patient 4 with a maximum of 165/105 mmHg and in patient 3 with maxima of 151 mmHg systolic and 107 mmHg diastolic. Arterial hypertension is known in the former and suspected in the latter, and the measurement before the start of the infusion already yielded hypertensive values similar to the ones during and after the infusion in both cases. Hence, a causal link between the medication and the hypertensive blood pressure seems unlikely. No abnormalities were documented for casirivimab/imdevimab infusions in the other two patients.

When the prevalence of the SARS-CoV-2 Omicron variant exceeded the Delta variant, we discussed a treatment switch to sotrovimab with our patients. Continued treatment with casirivimab/imdevimab was likely to become inefficacious for prophylaxis of SARS-CoV-2 infection in this context since the antibody cocktail’s neutralizing activity against the Omicron strain had been demonstrated to be insufficient. As the European Commission granted marketing authorization for sotrovimab only for the treatment of early COVID-19, infusions had to be administered off-label for pre-exposure prophylaxis in our patients. All of them were in favor of the treatment switch. The first infusions with 500 mg sotrovimab were conducted four weeks after the last casirivimab/imdevimab infusion and took place between 7^th^ and 11^th^ of February, 2022. Hypertensive blood pressure was measured before, during, and after the infusion in patients 3, 4, and 5. Again, as the blood pressure was already high in all patients before the start of the infusion, a causal link to the infusion is unlikely. No other adverse events were recorded. Due to its longer half-life compared to casirivimab/imdevimab, sotrovimab was planned to be administered every eight weeks at a dose of 500 mg.

At the end of March, 2022, the antibody cocktail tixagevimab/cilgavimab received marketing authorization in the European Union. Because this antibody combination was shown to have retained, albeit reduced, neutralizing activity against the SARS-CoV-2 Omicron strain and was approved specifically for pre-exposure prophylaxis, we conducted another treatment switch. Again, all patients approved of the switch and received their first injections eight weeks after their first sotrovimab infusion. No adverse events were recorded for tixagevimab/cilgavimab. Subject to the epidemiological situation, the next tixagevimab/cilgavimab injections are planned six months after the first dose.

The applied pre-exposure prophylactic treatment schedules for the different SARS-CoV-2-neutralizing monoclonal antibodies in our patients, the antibodies’ marketing authorization status in the EU for pre-exposure prophylactic treatment, and their neutralizing capacity against the SARS-CoV-2 Omicron strain are summarized in **Table 3**.

**Table 3:** Pre-exposure prophylactic treatment schedules for the different SARS-CoV-2-neutralizing monoclonal antibodies in our patients, status of marketing authorization for pre-exposure prophylactic treatment in the EU, and neutralizing capacity against the SARS-CoV-2 Omicron strain.

SARS-CoV-2-neutralizing antibodies	Casirivimab/Imdevimab	Sotrovimab	Tixagevimab/Cilgavimab
Route of application	Single intravenous infusion or subcutaneous injection	Intravenous infusion	Two separate intramuscular injections
Dose	First treatment 600 mg/600 mg Repeat treatment 300 mg/300 mg	500 mg	150 mg/150 mg
Treatment interval	Every four weeks	Every eight weeks	Every six months
Approved for pre-exposure prophylaxis in EU	+	–	+
Neutralizing capacity against SARS-CoV-2 Omicron strain	None or insufficient	Fully or largely retained	Retained, but reduced

+, approved for pre-exposure prophylaxis in EU; -, not approved for pre-exposure prophylaxis in EU.

Patients were regularly asked at routine clinical visits about occurrence of symptoms suggestive of an upper respiratory tract infection or positive SARS-CoV-2 testing. Data collected until the 2^nd^ of June, 2022, were taken into account for this report, corresponding to a follow-up time between 139 and 168 days since the first neutralizing antibody infusion. Patient 1 had a positive RT-PCR for SARS-CoV-2 on the 8^th^ of March, 2022, but was asymptomatic. His last sotrovimab infusion had taken place on the 10^th^ of February. Unfortunately, data on the SARS-CoV-2 variant are not available. The five other patients did not develop symptoms of an upper respiratory tract infection or had a positive antigen or RT-PCR test for SARS-CoV-2 during follow-up.

## Discussion

To our knowledge, no case reports on the use of monoclonal neutralizing antibodies for pre-exposure prophylaxis of SARS-CoV-2 infection in MS patients have been published yet. As mentioned above, several German Medical Societies recommend to consider treatment with SARS-CoV-2-neutralizing antibodies for patients who have an increased risk for severe COVID-19, for example due to immunotherapy, and who do not mount adequate immune responses to vaccination (3). These criteria are fulfilled by a relevant number of MS patients, especially those receiving immunomodulatory treatment with S1PR modulators or anti-CD20 antibodies. In order to identify affected individuals, it is necessary to screen MS patients on these disease-modifying therapies for immune responses to SARS-CoV-2 vaccination. In our Center, we conduct a screening not only for humoral, but also for T-cellular responses to the SARS-CoV-2 spike protein. The underlying rationale is that T-cells are able to confer protection against SARS-CoV-2 infection as well and that some patients, especially those on B-cell-depleting therapy, develop poor antibody, but good or even enhanced T-cellular responses to SARS-CoV-2 vaccination (24–26). We considered neutralizing antibody treatment only for those patients who lacked both humoral and T-cellular responses to SARS-CoV-2 after three doses of vaccination.

Treatment with S1PR modulators itself does not seem to increase the risk for severe COVID-19 disease (27–29). However, the lacking immune response to active SARS-CoV-2 vaccination caused by the S1PR modulator treatment does constitute a risk factor, and most of our patients who were treated with SARS-CoV-2-neutralizing antibodies had additional risk factors for severe COVID-19 beyond the lacking vaccination response. Patient 6, however, was 44 years old and did not have relevant comorbidities. Further, she had a low EDSS of 1.5 corresponding to no relevant neurological disability. She did have an increased risk of contracting SARS-CoV-2 because of the limited feasibility to reduce social contacts due to her three school-age children. According to the summary of product characteristics of casirivimab/imdevimab and tixagevimab/cilgavimab, their prophylactic use is not limited to defined groups of patients with certain risk factors so that treatment of patient 6 with these antibodies was possible within the marketing authorization. The patient felt much more secure on neutralizing antibody treatment which had a noticeable effect on her quality of life. On the other hand, it should be noted that several patients in our MS Center had similar constellations encompassing immunomodulatory treatment, a lack of vaccination responses, and no additional risk factors for severe COVID-19, and they were not treated with SARS-CoV-2-neutralizing antibodies. Treatment decisions need to be adapted to each patient’s situation, in consideration of the individual risk profile for severe COVID-19 and the degree of exposure. Generally, MS patients lacking an immune response to active vaccination, but without any risk factors for severe COVID-19 or increased exposure, likely do not need pre-exposure prophylactic antibody treatment. This especially applies when the Omicron variant is the predominant circulating strain as it usually only causes mild disease.

For casirivimab/imdevimab, published data from clinical studies show that its application is efficacious and safe not only for the treatment of COVID-19 disease, but also for the prophylaxis of SARS-CoV-2 infection (5–9). An *in vitro* study showed potent neutralizing activity for the antibody cocktail against Delta virus-like particles making it a suitable treatment as long as the SARS-CoV-2 Delta variant was the predominant viral strain (11). However, no neutralizing capacity of casirivimab/imdevimab against Omicron virus-like particles was detected (11). Another *in vitro* study demonstrated that the Omicron spike protein is completely resistant to imdevimab and mostly resistant to casirivimab (10). Thus, casirivimab/imdevimab became an unsuitable treatment when the incidence of infections with the Omicron variant was rising. For sotrovimab, by contrast, two *in vitro* studies demonstrated sustained neutralizing capacity against the SARS-CoV-2 Omicron variant (10, 15). In another analysis, the neutralizing capacity against an infectious SARS-CoV-2 Omicron isolate was only marginally reduced for sotrovimab’s parent antibody S309 (16). S309 had originally been derived from memory B cells of a convalescent individual infected with SARS-CoV in 2003. It binds a proteoglycan epitope on the viral spike protein distinct from the receptor-binding motif. The targeted epitope is highly conserved among sarbecoviruses explaining the antibody’s ability to neutralize not only SARS-CoV, but also SARS-CoV-2 including its known variants (30). Therefore, we decided to switch our patients’ pre-exposure prophylactic treatment to sotrovimab when the SARS-CoV-2 Omicron strain became prevalent. Because sotrovimab had not been granted marketing authorization by the European Commission for prophylactic use due to limited data on its efficacy in this context, the infusions remained an off-label treatment. Nonetheless, we assessed the treatment switch as the more sensible decision in the face of absent neutralizing activity of casirivimab/imdevimab against the SARS-CoV-2 Omicron strain. Tixagevimab/cilgavimab was the first monoclonal antibody combination with retained neutralizing activity against the Omicron variant approved for pre-exposure prophylaxis in the European Union. Treatment was hence switched in our patients as soon as this in-label treatment option was available. However, neutralizing capacity against the Omicron strain was shown to be reduced for tixagevimab/cilgavimab *in vitro* and also for sera obtained from immunocompromised patients after treatment with the antibody cocktail (19–23). It was suggested that higher treatment doses might be necessary in order to reach a greater degree of neutralization in the serum of treated patients. More data are needed in order to optimize treatment doses and to evaluate clinical efficacy.

Clearly, an intrinsic immune response to active immunization is favorable over continuous passive immunization. However, all patients reported here were not able to mount an immune response to vaccination with BNT162b2, mRNA-1273, and/or AZD1222. In the meantime, the European Commission has granted conditional marketing authorization for the protein-based adjuvanted vaccine Nuvaxovid (Novavax CZ). We recommend our S1PR modulator-treated patients lacking immune responses to SARS-CoV-2 mRNA and/or vector vaccination to get vaccinated with Nuvaxovid, now that it is available in Germany. Possibly, this vaccine will be more effective in eliciting an immune response in the respective patients due to the included immune adjuvant. If so, it will be possible to discontinue neutralizing antibody treatment.

In this case series, we present six MS patients who received pre-exposure prophylactic treatment with SARS-CoV-2-neutralizing antibodies due to their inability to mount an immune response to active SARS-CoV-2 vaccination on account of their immunomodulatory treatment. In times of a predominance of the SARS-CoV-2 Delta variant, casirivimab/imdevimab was a suitable treatment option which is authorized for prophylactic use. In times of higher incidences of the Omicron variant, we considered treatment with sotrovimab to be more suitable, but this antibody had to be administered off-label as no sufficient data on its prophylactic use are available yet. Tixagevimab/cilgavimab is the first monoclonal antibody combination approved for pre-exposure prophylaxis in the European Union with sustained neutralizing activity against the Omicron strain. In our opinion, it thus seems to be the best treatment option in patients who need pre-exposure prophylactic SARS-CoV-2-neutralizing antibody treatment in times of high incidences of infections with the Omicron variant. One patient in our case series had a positive RT-PCR for SARS-CoV-2 under sotrovimab treatment, but was asymptomatic. For the other patients, no symptoms typical of COVID-19 and no evidence of SARS-CoV-2 infection were recorded during the follow-up of 139 to 168 days under neutralizing antibody treatment. Importantly, we did not observe any significant adverse events. Neutralizing antibody treatment remains a treatment option that needs evaluation for and discussion with each individual patient according to their risk profile and individual preference. Of course, deductions on the efficacy and safety of SARS-CoV-2-neutralizing antibody treatment cannot be made from this case series. Hopefully, immunization with adjuvanted protein vaccines will be able to elicit adequate immune responses also in S1PR modulator-treated MS patients rendering neutralizing antibody treatment for pre-exposure prophylaxis of SARS-CoV-2 infection unnecessary.

## Data availability statement

The original contributions presented in the study are included in the article/supplementary material. Further inquiries can be directed to the corresponding author.

## Ethics statement

The studies involving human participants were reviewed and approved by Ethikkommission an der Technischen Universität Dresden. The patients/participants provided their written informed consent to participate in this study. Written informed consent was obtained from the individual(s) for the publication of any potentially identifiable images or data included in this article.

## Author contributions

Conception and design: CW, KA, and TZ. Writing the manuscript: CW. Creation of figure and tables: CW. Critical feedback and clinical management: UK. Revision for important intellectual content: KA and TZ. All authors approved the final version to be published.

## Conflict of interest

CW received travel support from Novartis. KA received personal compensation from Roche, Sanofi, Teva, Merck, Alexion, BMS, and Celgene for oral presentations and consulting services. TZ received personal compensation from Biogen, BMS, Bayer, Merck, Novartis, Roche, Sanofi, Teva, and Viatris for consulting and speaking services. TZ received additional financial support for research activities from Biogen, Novartis, Roche, Teva, and Sanofi. TZ is principal investigator of the AMA-VAC and KYRIOS study.

The remaining author declares that the research was conducted in the absence of any commercial or financial relationships that could be construed as a potential conflict of interest.

## Publisher’s note

All claims expressed in this article are solely those of the authors and do not necessarily represent those of their affiliated organizations, or those of the publisher, the editors and the reviewers. Any product that may be evaluated in this article, or claim that may be made by its manufacturer, is not guaranteed or endorsed by the publisher.
